# Experimental Investigation of Effect of Flake Silver Powder Content on Sintering Structure and Properties of Front Silver Paste of Silicon Solar Cell

**DOI:** 10.3390/ma15207142

**Published:** 2022-10-13

**Authors:** Wei Li, Chunxiu Yu, Yunkai Wang, Yuan Yao, Xianglei Yu, Chuan Zuo, Yang Yu

**Affiliations:** 1 Sino-Platinum Metals Co., Ltd., Kunming 650106, China; 2 Key Laboratory of Advanced Materials of Yunnan Province, School of Material Science and Engineering, Kunming University of Science and Technology, Kunming 650093, China; 3 Centre for Infrastructure Engineering, Western Sydney University, Penrith, NSW 2751, Australia

**Keywords:** front silver paste, sintering activity, morphology of silver powders, silver film, optimal ratio, flake silver powder

## Abstract

Optimizing the performance of front silver paste is of great significance in improving the efficiency of the photoelectric conversion of crystalline silicon solar cells. As a conductive functional phase of silver paste, the structure and performance of silver powder have an important influence on the sintering process of silver paste and the conductivity of silver electrodes. Because of their two−dimensional structure, flake silver powders can effectively increase the contact area with other silver powders and silicon cells before sintering. Additionally, flake silver particles have higher surface energy and sintering activity than spherical silver particles of the same particle size. However, recent research has mainly focused on the influence of the particle size of silver powder. This paper fills the research gap regarding the morphology of silver powders and clarifies the influence of flake silver powders on the performance of silver paste. The influence of the ratio of spherical silver powder to flake silver powder in silver paste on the sheet resistance, adhesion, and specific contact resistivity of silver film after sintering at 800 °C was studied, and the optimal ratio was determined according to a cross−sectional contact picture of the silver film. The results showed that with the increase in the mass fraction of the flake silver powder, the sheet resistance of the sintered silver film gradually increased, the adhesion first increased and then decreased, and the specific contact resistance first decreased and then increased. When the flake silver powder content was 0%, the minimum sheet resistance of the silver film was 2.41 m Ω/☐. When the flake silver powder content was 30%, the maximum adhesion of the silver film was 6.07 N. When the flake silver powder content was 50%, the minimum specific contact resistivity of the silver film was 0.25 Ω·cm^2^. In conclusion, when the flake silver powder content was 30%, the comprehensive performance of the silver film was the best.

## 1. Introduction

With the goal of peak carbon dioxide emissions and carbon neutrality being implemented, the new energy revolution and the diversification of energy structures will be accelerated, and photovoltaic cells, as the center of new energy, will gradually occupy the main position. Among many photovoltaic cells, crystalline silicon solar cells occupy 90% of the world’s photovoltaic cell market share and are the most widely used photovoltaic cells. Therefore, many researchers have paid attention to their development prospects and market value [1,2,3,4]. Silver paste used for the front electrodes of solar cells is one of the key materials used in the production of crystalline silicon solar cells [5]. At present, due to the considerations of cost, it is generally printed on silicon cells coated with anti−reflection film using high−speed and high−resolution screen printing, and then the electrodes are formed after drying and high−temperature sintering [6,7]. The properties of the silver paste and sintering procedure in the electrode preparation procedure have a remarkable influence on the light−receiving area and the series resistance of solar cells [8,9]. The performance of the front silver paste plays a significant role in the photoelectric conversion rate of solar cells. The silver paste printing performance determines the width of the front electrode sub−grid line of the solar cell and directly affects the light−receiving area of solar cells [10]. The density and sheet resistance of silver film formed by sintering are important factors that affect the series resistance and filling factor of solar cells [11,12]. During the silver paste sintering process, the ability of glass melt to corrode the silicon nitride anti−reflection layer as well as its silver dissolution and precipitation abilities, are among the main factors that determine whether the silver film can form good ohmic contact with the silicon substrate. Additionally, these are the main factors that do not destroy the P–N junction of the solar cell itself [13,14].

To reduce the cost of solar cells and to improve the photoelectric conversion efficiency, Ma et al. [15] and Pi et al. [16] studied the influence of glass frit on the resistivity, adhesion, and microstructure of front silver paste by improving the glass formulation, and prepared a glass frit with better thermal stability (low dissolution rate and low thermal expansion coefficient and softening temperature), which was able to wet the Ag powder and construct a uniform electrode, effectively improving the photoelectric conversion efficiency of solar cells. The highest photoelectric conversion rate of the front silver paste prepared by Ma was 18.260%, and the lowest series resistance was 0.0017 Ω. Rane et al. [17], Yang et al. [5], and Qin et al. [18] studied the sintering process of front silver paste and the contact formation process of silver electrodes and silicon wafers, and analyzed the influence of silver electrodes and the microstructure of the silver–silicon interface formed by different silver pastes on the adhesion, contact resistance, and photoelectric properties of the solar cells. Rane studied the effects of glass powder content and silver powder surface treatment on the structure and properties of silver electrodes, and the lowest bulk resistivity of the silver electrodes was found to be 7.2 × 10^−6^ Ω cm. Yang studied the Ag–Si microstructure of different commercial silver pastes, and found that the adhesion of the silver electrodes could affect the service life and reliability of the battery, and the decrease in adhesion can reduce the output power of the battery; the highest adhesion was more than 3.0 N/1.6 mm. Qin studied the influence of different glasses on the sintering process, sintering compactness, and sintering interface, and the contact resistance of the silver electrodes was between 1 and 2 Ω. Li et al. [19], Wang et al. [20], and Mo et al. [21] systematically studied the effects of the micron silver powder, submicron silver powder, and silver paste of nano−silver powder on the contact interface structure, the contact interface performance, and the performance of the solar cells. Li found that the surface sintering temperature of the nano−silver powder was low, which could increase the silver ion content in the glass melt to form a continuous silver layer on the silicon interface and could also reduce the specific contact resistivity of the battery, which was about 0.0052 Ω cm^2^. Wang studied the effect of mixing micron silver powder and submicron silver powder on the contact interface structure and ohmic contact performance; the highest photoelectric conversion rate was found to be 18.282%, and the lowest series resistance was 0.0019 Ω. Mo studied the effect of adding submicron silver powder on the morphology and compactness of a silver electrode fine grid; the highest photoelectric conversion rate, the lowest series resistance, and the lowest specific ohmic contact resistance of the prepared silver paste were 17.44%, 0.00314 Ω, and 0.015 Ω cm^2^, respectively. In summary, the research on silver powder for solar front silver paste has mainly focused on the particle size of the silver powder, and there is a lack of research on the electrode structure and interface contact performance after silver paste sintering with the addition of flake silver powder. Because of its two−dimensional structure, flake silver powders can form a linear contact or surface contact with other silver powders and silicon cells before sintering, which effectively increases the contact area and improves the contact performance. Moreover, flake silver particles have a larger specific surface area, larger surface energy, and better surface sintering activity than spherical silver particles of the same particle size. It is expected that the addition of flake silver powder can improve the sintering performance of silver paste to form a denser silver electrode and better silver–silicon interface contact.

Reducing the thickness of crystalline silicon cells and simultaneously using low−concentration shallow junction diffusion processes is an effective method by which to improve the photoelectric conversion efficiency of solar cells, but this method has higher requirements for the sintering process and subsequent silver paste performance. Under the low−temperature and fast−sintering process (here, this refers to a low temperature within the defined high−temperature range), we studied the change process of the flake silver powder and mixed silver powder in a solar high−temperature silver paste during the sintering process. It is of great research significance to clarify the sintering activity of the flake silver powder and a suitable sintering process. It is also of great significance to broaden the available types of silver powder in high−temperature silver paste and to improve the electrical and mechanical properties of the silver film after sintering the solar front silver paste as well as to reduce the overall resistance of solar cells, increase the short−circuit current, and increase the filling factor. Therefore, we mainly studied the changes in spherical silver powder, flake silver powder, and mixed silver powder in the sintering process of silver paste on the front of the solar cells. We also investigated the effects of different proportions of flake silver powder and spherical silver powder on the electrical properties and adhesion of silver films by studying and optimizing the composition and sintering process of silver paste to satisfy the continuous improvement performance requirements of front silver paste in new solar cells.

## 2. Materials and Methods

### 2.1. Experimental Materials

**Figure 1:**
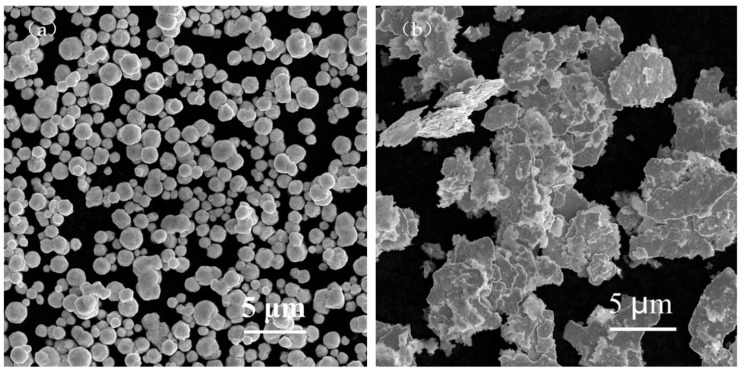
SEM images of the spherical silver powder and flake silver powder. (**a**) Spherical silver powder. (**b**) Flake silver powder.

**Figure 2:**
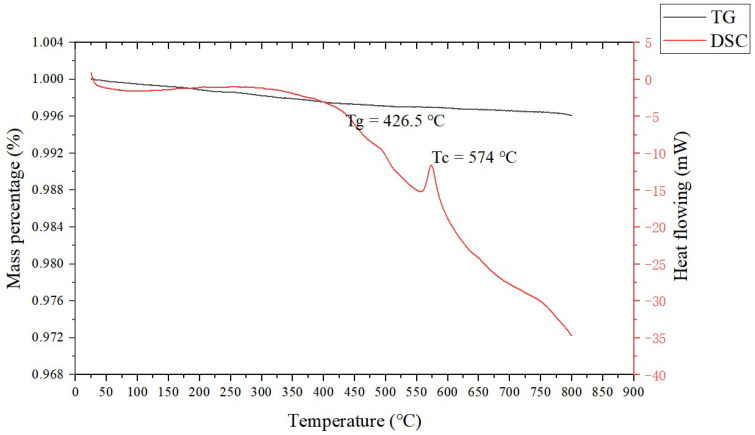
DSC chart of the glass powder.

The raw materials included spherical silver powder with an average particle size of 1.3 µm (as shown in Figure 1a) and flake silver powder with a mean particle size of 5.8 µm (as shown in Figure 1b). A DSC picture of the Bi_2_O_3_−B_2_O_3_−SiO_2_−system lead−free glass powder is shown in Figure 2. The transition temperature (Tg) of the glass powder was 426.5 °C. The crystallization temperature (TC) of the glass powder was 574 °C. For the organic carrier, the mass percentage of each component was as follows: terpineol 55%, butyl carbitol 20%, dibutyl phthalate 11.1%, silane coupling agent (KH−5700) 3.1%, lecithin 0.3%, polyamide wax slurry 0.5%, ethyl cellulose ether 5%, and acrylic resin 5%. A coating with silicon nitride anti−reflection coating and a monocrystalline silicon substrate was used. Analytical pure anhydrous ethanol, 101−type environmental protection flux, and Pb40/Sn60 type welding tape were used.

### 2.2. Preparation and Sintering of Front Silver Paste for Solar Cells

The front silver paste was prepared by mixing 84% silver powder, 4.5% glass powder, 1% dopant, and 10.5% organic carrier, respectively. First, the solid powder was mixed uniformly in the mixer, then the organic carrier was added, and the semi−finished slurry was obtained by stirring uniformly. Finally, the slurry was repeatedly ground using a three−roll mill to form a front silver paste with a fineness of less than 10 µm.

The prepared front silver paste was printed on a single crystal silicon substrate coated with a silicon nitride anti−reflection film using a screen−printing machine to form electrode grid lines. Then, the silver paste was placed in a high−temperature chain sintering furnace, dried for 5 min at 250 °C, and then sintered at different peak temperatures to obtain the required solar electrodes.

### 2.3. Testing and Characterization

**Figure 3:**
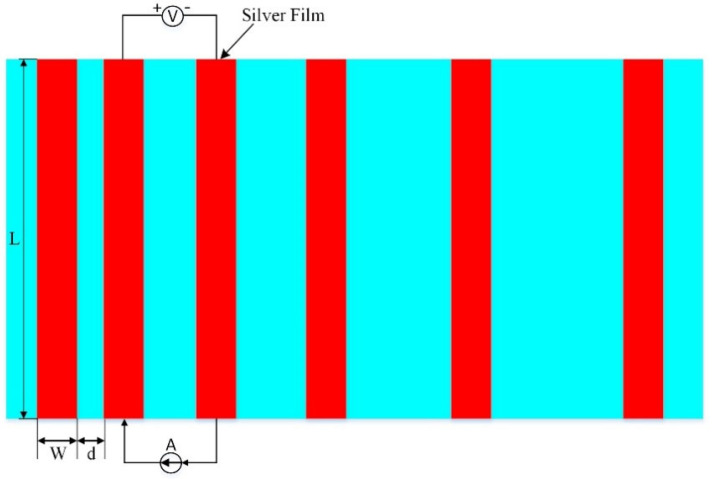
Schematic of the TLM method for the contact resistivity measurements. L = 20 mm, W = 1 mm, and d is from 0.5 mm to 2.5 mm with an increment of 0.5 mm.

The sintered silver film sheet resistance was tested using an ST−2258C multifunctional four−probe tester. XL30ESEM−TEP SEM was employed to analyze and observe the cross−sectional morphology and surface morphology of the sintered silver film. A silver paste with a size of 1.5 mm × 30 mm was printed on the monocrystalline silicon substrate and sintered according to the corresponding process, and then a 1 mm wide Pb40/Sn60−type solder tape was welded on the silver film at 340 °C using an intelligent electric welding station. The solder tape was first soaked in flux for 10 min, and, finally, the adhesion between the sintered silicon substrate and the silver film was tested by using a tensile machine. The ohmic contact resistivity between the silicon substrate and the silver film was measured using the rectangular transmission line method (TLM) as shown in Figure 3 [22,23].

A constant current I = 5 mA was passed between the two electrodes, and the current was restricted to flow between the parallel electrodes to obtain the resistance R between any two adjacent electrodes. The resistance R can be expressed as:

(1) $$R=2Rc+\frac{Rshd}{L}$$

where *R_c_* denotes the contact resistance between the electrode and semiconductor; *R_sh_* is the sheet resistance of the silicon−based semiconductor between the electrodes.

Equation (1) shows that the resistance *R* between two adjacent electrodes has a linear relationship with the electrode spacing *d*, and the intercepts of the straight line on the *R*−axis and the *d*−axis are 2*R_c_* and *d_t_*, respectively. The ohmic contact resistivity *ρ_c_* of the silicon substrate and silver film can be calculated by Equation (2):

(2) ρc=RcdtL

## 3. Results and Discussion

### 3.1. Sintering Changes of Different Silver Powders

**Figure 4:**
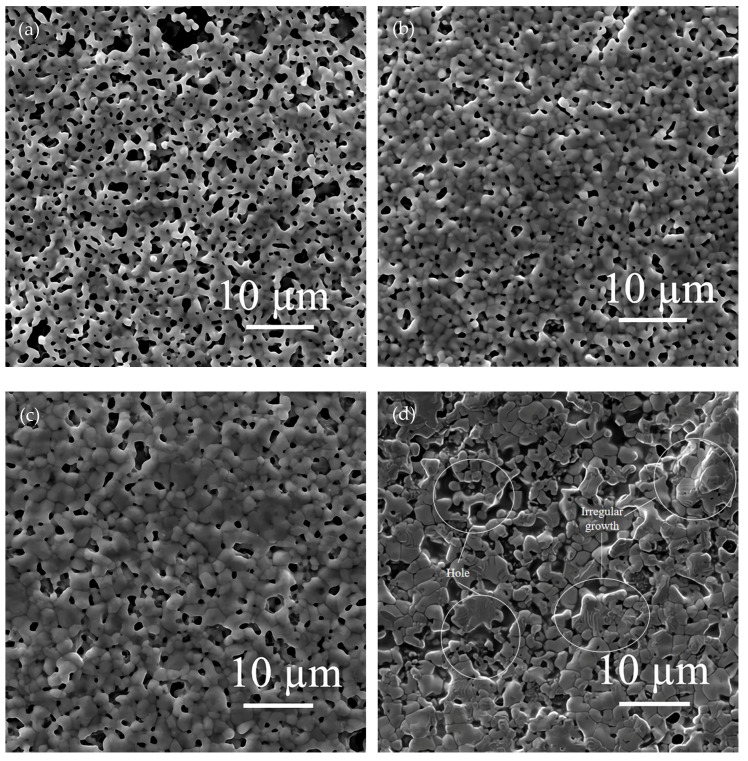
Surface morphology of sintered silver pastes at different temperatures. Images in (**a**–**d**) are for the silver paste with spherical silver powder sintered at 600, 700, 800, and 900 °C, respectively.

**Figure 5:**
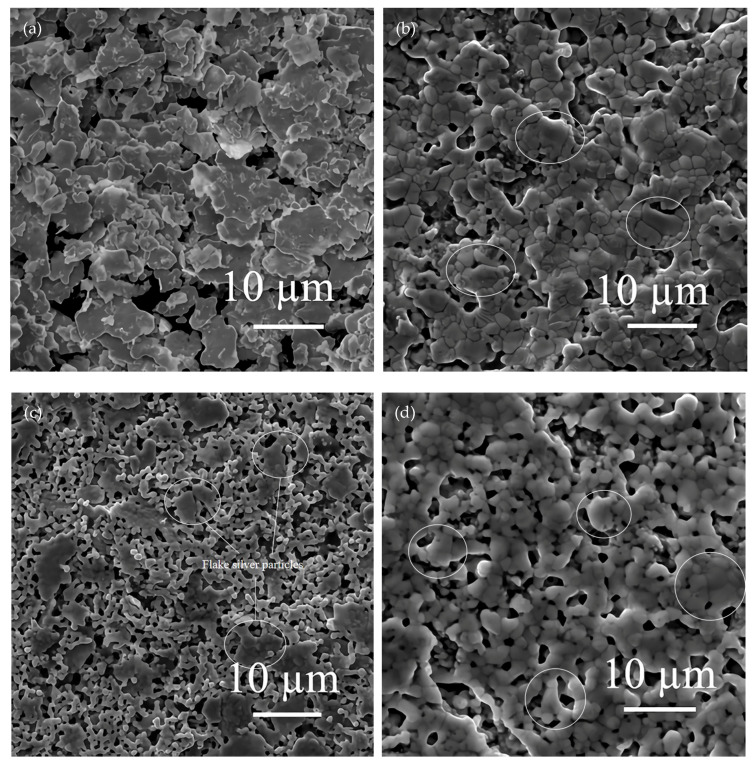
Surface morphology of sintered silver pastes at different temperatures. Images in (**a**,**b**) are for the silver paste with flake silver powder sintered at 600 and 800 °C, respectively. Images in (**c**,**d**) are for the silver paste with mixed silver powder sintered at 600 and 800 °C, respectively.

The silver paste was prepared with spherical silver powder with a mean particle size of 1.3 µm, flake silver powder with a mean particle size of 5.8 µm, and a mixed silver powder composed of two silver powders in a 1:1 mass ratio. All silver pastes were sintered at 600, 700, 800, and 900 °C for 40 s, respectively. The sintered samples were observed by SEM to analyze the changes in the silver paste during the sintering process. The SEM pictures are presented in Figure 4 and Figure 5.

By observing the changes in the sintering morphology of the silver paste prepared from spherical silver powder with an average particle size of 1.3 µm at different temperatures, the appropriate sintering temperature was confirmed. When the sintering temperature reached 600 °C, the organic carriers in the silver paste were basically decomposed and oxidatively discharged, leaving only the glass powder and silver powder to participate in the sintering, and the initial sintering state of the silver particles was easily observed. When the sintering temperature reached 900 °C, it was close to the melting point of silver at 961.93 °C. If the temperature exceeds 900 °C, it will be higher than the heat−treatment temperature (800–900 °C) when the solar cell is doped and diffused to form a P–N junction. Too high a sintering temperature will affect the performance of the solar cell. Therefore, four temperature points of 600, 700, 800, and 900 °C were selected for sintering for 40 s, and these temperatures were used to preliminarily study the change in spherical silver particles at different sintering temperatures. The SEM morphology of the silver film surface after silver paste sintering is shown in Figure 4.

According to the SEM picture shown in Figure 4a, although the sintering temperature exceeded the glass transition temperature of 426.5 °C, when the peak temperature reached 600 °C, the viscosity of the glass melt was not low enough, and the fluidity was poor, which led to connections only being formed between the silver particles that were close to each other; this did not allow the silver particles to rearrange themselves to form a compact conductive network. In Figure 4b,c, it can be noted that as with the increase in the peak temperature of the silver paste sintering, the viscosity of the glass melt decreased and the fluidity increased, which allowed for better infiltration of the battery substrate and silver powder and was more effective. This drove the rearrangement of the silver powder particles and filled the voids to establish a denser conductive network, thus reducing the sheet resistance of the silver film. On the other hand, by comparing Figure 4a–d, it can be seen that the particle size of the silver powder gradually increased with the increase in the peak sintering temperature of the silver paste, and, at this time, the sintering temperature of the silver paste was far lower than the melting temperature of silver, and the silver particles did not melt. This phenomenon occurred because the silver powder with a small particle size and high energy dissolved in the glass melt as the sintering temperature increased and diffused to the large size of the particles and low energy through the liquid phase mass transfer of the glass melt. The precipitation on the surface of the silver particles caused them to increase in size. When the peak temperature of the silver paste sintering was further increased to 900 °C, as shown in Figure 4d, because the sintering temperature was close to the melting point of silver, the silver particles that were close to each other melted together and grew irregularly. The dimensional shrinkage caused by the melting together formed a great number of holes in the conductive network, which reduced the conductivity of the silver film and increased the sheet resistance.

From the above analysis, it can be seen that the suitable sintering temperature for the silver paste was 800 °C. We selected 600 °C, at which the initial sintering state of silver paste was observed, and the most suitable temperature of 800 °C to sinter the silver paste made of pure flake silver powder and the silver paste made by mixing the flake silver powder and spherical silver powder at a ratio of 1:1, respectively. We observed the shape change of the flake silver particles after sintering. The surface morphology of the sintered silver film is shown in Figure 5.

According to the SEM pictures in Figure 5a,c, it can be seen that when the peak sintering temperature of the silver paste was 600 °C, the flake silver particles were arranged in a flat layer and did not change significantly after sintering. The main sintering process was that the glass powder softened and partially soaked the silver particles, and meanwhile, a connection between the silver particles was formed. When the peak sintering temperature of the silver paste was 800 °C, by comparing the SEM images of Figure 5a,b, it can be seen that during the sintering process, the glass melt soaked the surface of the flake silver particles, and the flake silver particles underwent internal polycondensation and transformed into spherical silver particles. This may have been due to the high energy at the edge of the flake silver particles during the sintering process. The silver at the edge of the particle was easily dissolved in the glass melt and diffused through the glass melt to the middle of the flake silver particles with lower energy, and then precipitated. With the continuous occurrence of this process, the flake silver particles continued to shrink and deformed into a spherical shape. However, it can be seen from the circled part in Figure 5b that the flake silver particle had not completely deformed into a spherical silver particle under the sintering condition of 800 °C for 40 s, and the shape of the silver particle was irregular and its size was large. Comparing Figure 4c and Figure 5b, we could see that the conductive network formed by the sintering of the flake silver powder was layered, and the conductive network formed by the sintering of the spherical silver powder was reticulated. Although the conductive network connection of the single layer of flake silver powder was denser and better than that of the spherical silver powder, the connection between the layers was not tight enough, which affected the conductivity of the sintered silver film. Comparing Figure 4c and Figure 5b,d, it can be seen that the sintering condition of the mixed silver powder was between that of the pure spherical silver powder and the pure flake silver powder, and the flake silver particles in the mixed silver powder shrank and the surrounding silver particles formed a small lamellar structure. Additionally, in terms of the size shrinkage of the flake silver powder, the pores formed by sintering the silver paste of the mixed silver powder were greater in number than those formed by sintering the pure spherical silver powder, and fewer than those of the pure flake silver powder.

In summary, the most suitable sintering temperature for silver paste was found to be 800 °C, and the three silver pastes were able to form a dense conductive network when sintered at this temperature for 40 s. Under this condition, some of the flake silver particles will shrink into spherical silver particles after sintering, but some of the flake silver particles will not become completely spherical, forming a layered structure with the surrounding silver particles. The conductive network in a layer is much denser, but the connection between layers is not tight.

### 3.2. Effect of Flake Silver Powder Content in Silver Paste on the Properties of Sintered Silver Film

**Figure 6:**
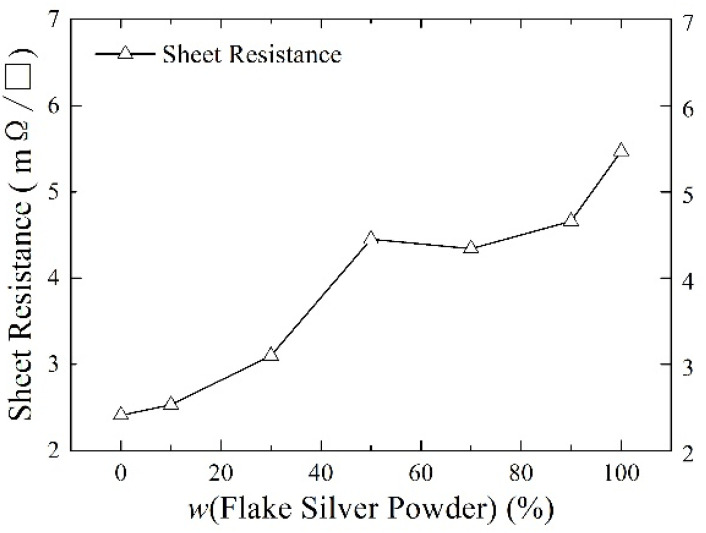
The effect of the flake silver powder content on the sheet resistance of the sintered silver film.

**Figure 7:**
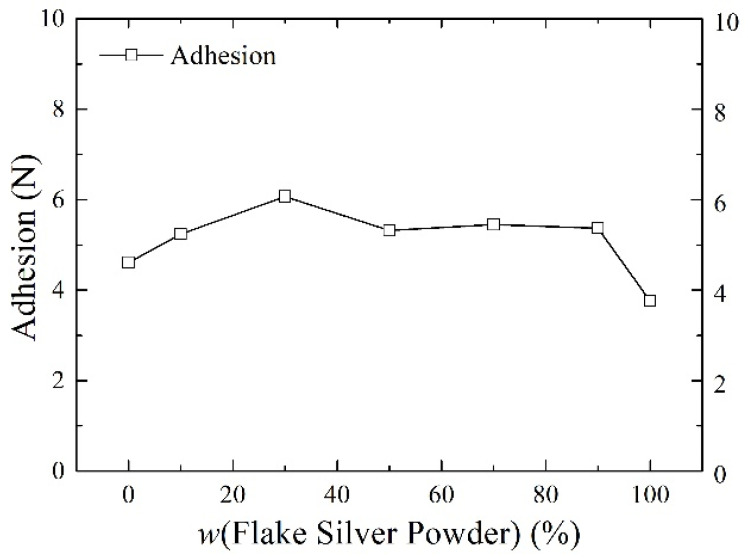
The effect of the flake silver powder content on the adhesion of the sintered silver film.

**Figure 8:**
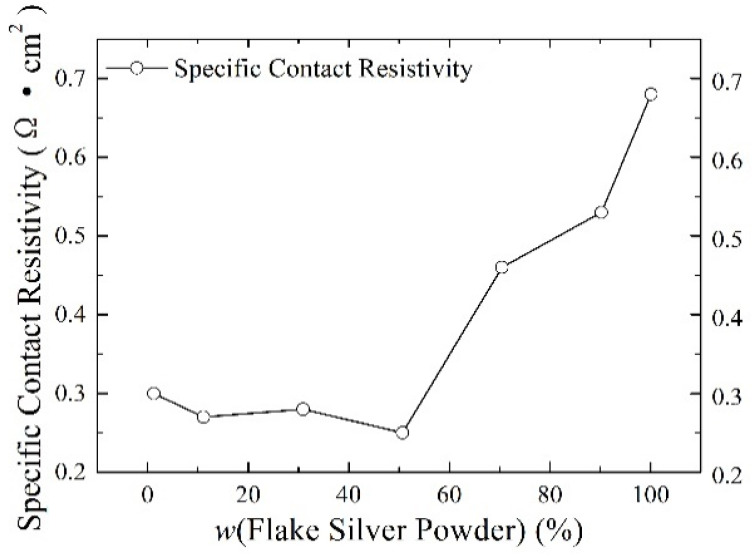
The effect of the flake silver powder content on the specific contact resistivity of the sintered silver film.

Silver paste was prepared from silver powder mixed with the flake silver powder and spherical silver powder according to different mass percentages and sintered at the peak temperature of 800 °C. The square resistance, adhesion to the silicon substrate, and specific contact resistivity of the sintered silver film were measured. The relationship between the content of the flake silver powder and the silver film performance is shown in Figure 6, Figure 7 and Figure 8.

#### 3.2.1. Effect of Flake Silver Powder Content on Sheet Resistance

As shown in Figure 6, the relationship between the content of the flake silver powder and the sheet resistance of the sintered silver film showed that with the increase in the content of the flake silver powder, the sheet resistance of the silver film after sintering increased continuously from 2.41 m Ω/☐ of pure spherical silver powder to 5.47 m Ω/☐ of pure flake silver powder. This is because a small amount of silver can be dissolved in the glass melt during the sintering process at medium and high temperatures, so the silver can carry out liquid phase mass transfer through the glass melt. The energy at the edge of the flake silver powder was higher, and it diffused to the middle part with lower energy through the glass melt, so the flake silver powder gradually shrank to a spherical shape. Since the sintered silver paste is not like the low−temperature curing paste, the flake silver powder will shrink during the sintering process, and the formation of a greater number of holes will affect the compactness of the sintered silver film and increase the sheet resistance of the silver film.

#### 3.2.2. Effect of Flake Silver Powder Content on the Adhesion

As shown in Figure 7, the relationship between the content of the flake silver powder and the adhesion between the silicon substrate and silver film showed that, with the increase in the content of flake silver powder in the silver paste, the adhesion of the silver film to the silicon substrate first increased and then decreased. When the flake silver powder accounted for 30% of the mass of the silver powder, the adhesion reached the maximum value of 6.07 N, and when it is reduced to pure flake silver powder, the adhesion reached the minimum value of 3.73 N. This is because the operating functions of silver and silicon are quite different, and direct contact between the silicon substrate and silver is not easily formed, with the glass melt usually playing a connecting role. Due to the high energy at the edge of the flake silver powder, despite most silver easily dissolving in the glass melt, part of the silver will be oxidized into the glass melt in the form of Ag^+^ ions, and this part of the Ag^+^ ions can reduce the viscosity of the glass melt and increase the flow. On the other hand, it can react with the silicon nitride anti−reflection layer on the surface of the silicon−based cell according to Equation (3) [19], which can help corrode the anti−reflection layer, allowing the glass melt and the silicon substrate to form a fuller contact, thereby improving the silver film. Therefore, when the content of flake silver powder is low, the adhesion between the silver film and silicon substrate after sintering increases with the increase in the flake silver powder content, while with the further increase in the flake silver powder content, the silver and silver ions dissolved in the glass melt become nearly saturated, which has no obvious influence on the adhesion. Additionally, due to the size shrinkage of the flake silver powder in the sintering process, with the increase in the flake silver powder content, the shrinkage of the whole silver paste during the sintering procedure intensifies, the gap between the sintered silver film and silicon substrate increases, and the adhesion between the silicon substrate and sintered silver film decreases.

(3) $$4Ag^{+}+2O^{2-}+SiNX\to 4Ag+SiO_{2}+x/2N_{2}$$

#### 3.2.3. Effect of Flake Silver Powder Content on the Specific Contact Resistivity

As shown in Figure 8, the relationship between the content of the flake silver powder and the specific contact resistance of the silver film and silicon substrate showed that with the increase in the content of flake silver powder in the silver paste, the specific ohmic contact resistance between the silver film and silicon substrate after sintering first decreased and then increased. When the flake silver powder accounted for 50% of the mass of the silver powder, the specific contact resistance reached the minimum value of 0.25 Ω·cm^2^, and when it increased for the pure flake silver powder, the specific contact resistance reached the maximum value of 0.68 Ω·cm^2^. The main reason contributing to this phenomenon is that with the increase in the flake silver powder content, the content of Ag+ ions and silver in the glass melt increases, and the Ag+ ions can react with the silicon of the silicon substrate, as demonstrated in Equation (4). After the glass melt contacts the silicon substrate [19], silver particles are formed on the surface of the silicon substrate. Additionally, the silver dissolved in the glass melt will also be precipitated during the cooling process. The silver precipitated at the contact between the silicon substrate and the silver film is partly deposited on the surface of the silicon substrate and the other part remains in the glass phase, which reduces the specific contact resistivity between the silicon substrate and silver film. When the content of the flake silver powder is small, the specific contact resistivity of the sintered silver film and silicon substrate decreases with the increase in the flake silver powder content. When the flake silver powder content is high, with the increase in the flake silver powder content, the gap will increase and the contact area will decrease between the silicon substrate and the silver film after sintering, which plays a crucial part in the change in the specific contact resistivity of the silver film, and the specific contact resistivity between the silicon substrate and silver film will increase.

(4) $$4Ag^{+}+2O^{2-}+Si\to 4Ag+SiO_{2}$$

#### 3.2.4. Micromorphology of the Contact Section between the Silicon Substrate and Silver Film

**Figure 9:**
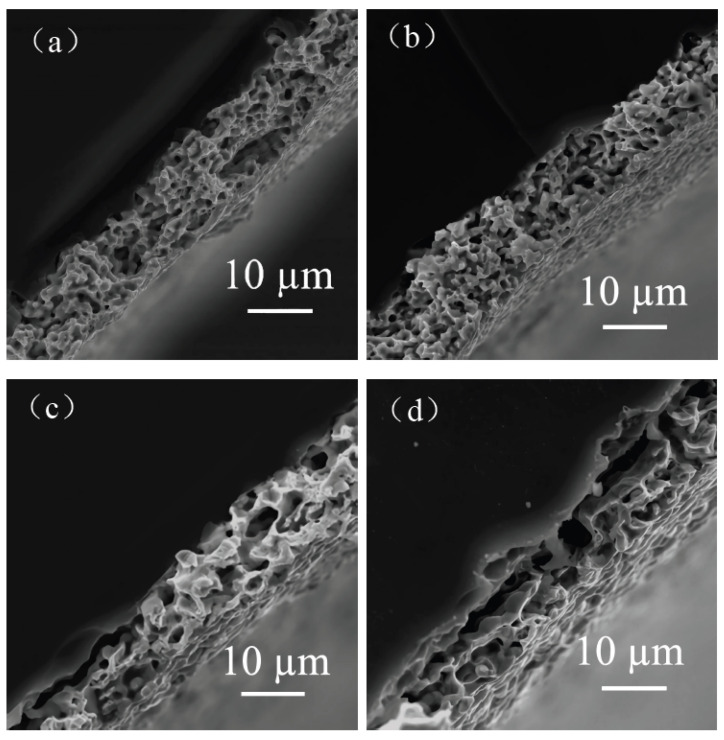
Cross−sectional view of contact between the silver film of the flake silver powder with different contents and silicon substrate. Image (**a**) is a cross−section of the contact between the silicon substrate and silver film with 0 wt% flake silver powder. Image (**b**) is a cross−section of the contact between the silicon substrate and silver film with 30 wt% flake silver powder. Image (**c**) is a cross−section of the contact between the silicon substrate and silver film with 50 wt% flake silver powder. Image (**d**) is a cross−section of the contact between the silicon substrate and silver film with 100 wt% flake silver powder.

The effects of different flake silver powder contents on the sheet resistance, adhesion, and specific contact resistivity of the sintered silver film were comprehensively compared. It was found that when the flake silver powder accounted for 30% and 50% of the mass of the mixed silver powder, the silver film performance was better. The micromorphology of the contact area between the silicon substrate and silver film with the flake silver powder accounting for 0%, 30%, 50%, and 100% of the mass of the silver powder was observed and analyzed by SEM. The SEM picture is presented in Figure 9.

It can be observed from the SEM pictures shown in Figure 9 that when the flake silver powder content was 30%, the pores of the silver film were the smallest, the contact area between the silicon substrate and silver film was large, and the adhesion was the tightest. When the flake silver powder content was 50%, the pores of the silver film became larger, and the gap between the silver film and silicon substrate increased. Combined with Figure 6, the sheet resistance of the silver film increased slowly when the flake silver powder content was between 0% and 30%, but increased rapidly when it was between 30% and 50%. Therefore, when the flake silver powder accounted for 30% of the mass of the mixed silver powder, the performance of the silver film was the best.

## 4. Conclusions

Silver pastes prepared from spherical silver powder, flake silver powder, and mixed silver powder were sintered under different peak temperature conditions. According to the results of our comparative analysis, the major outcomes can be summarized as follows:

(1) The viscosity of the glass melt decreased and the fluidity increased at a peak sintering temperature of 800 °C, allowing it to quickly infiltrate the silver particles and drive the rearrangement of the silver particles to establish a dense conductive network, thus improving the silver film conductivity.
(2) When the sintering peak temperature of the silver paste was 800 °C, the flake silver particles were partially transformed into spherical silver particles and shrank in size. A denser layered conductive network was easily formed around the flake silver particles. However, due to the size shrinkage of the flake silver particles, a large number of holes were left between the layers, which affected the conductivity. When the peak sintering temperature of silver paste reached 900 °C, the silver particles that were in contact with each other melted, resulting in a large number of holes, which affected the silver film’s conductivity.
(3) Due to the size shrinkage of the flake silver powder during sintering being greater than that of the spherical silver powder, the addition of flake silver powder increased the porosity between the silver electrode sheets and increased the sheet resistance of silver electrodes. However, when the flake silver powder content was low, the flake silver powder increased the contact area at the silver–silicon interface, and the high sintering activity of the flake silver powder was found to be beneficial to the formation of higher bonding strengths at the silver–silicon interface, which increased the adhesion and reduced the contact resistance. Additionally, because the contact resistance in the series resistance of the battery was much higher than the sheet resistance, the addition of a small amount of flake silver powder was found to be beneficial to the sintering performance of the silver paste and the performance improvement of solar cells. It was found that the comprehensive performance of the silver film was the best when the flake silver powder content was 30%.

## Data Availability

Not applicable.
